# Reporting of Morphology, Location, and Size in the Treatment of Osteochondral Lesions of the Talus in 11,785 Patients: A Systematic Review and Meta-Analysis

**DOI:** 10.1177/19476035241229026

**Published:** 2024-02-16

**Authors:** Pascal R. van Diepen, Frank F. Smithuis, Julian J. Hollander, Jari Dahmen, Kaj S. Emanuel, Sjoerd A.S. Stufkens, Gino M.M.J. Kerkhoffs

**Affiliations:** 1 Department of Orthopaedic Surgery and Sports Medicine, Amsterdam UMC, Location AMC, University of Amsterdam, Amsterdam, The Netherlands; 2 Academic Center for Evidence Based Sports Medicine (ACES), Amsterdam UMC, Amsterdam, The Netherlands; 3 Amsterdam Collaboration for Health and Safety in Sports (ACHSS), International Olympic Committee(IOC) Research Center, Amsterdam UMC, Amsterdam, The Netherlands; 4 Amsterdam Movement Sciences, Programs Sports and Musculoskeletal Health, Amsterdam, The Netherlands; 5 Department of Radiology and Nuclear Medicine, Amsterdam UMC, Location AMC, University of Amsterdam, Amsterdam, The Netherlands

**Keywords:** osteochondral lesions, talus, ankle, cartilage repair, reportage

## Abstract

**Objective:**

Uniformity of reporting is a requisite to be able to compare results of clinical studies on the treatment of osteochondral lesions of the talus (OLT). The primary aim of this study was to evaluate the frequency and quality of reporting of size, morphology, and location of OLTs.

**Design:**

A literature search was performed from 1996 to 2023 to identify clinical studies on surgical treatment of OLTs. Screening was performed by 2 reviewers, who subsequently graded the quality using the methodological index for non-randomized studies (MINORS). The primary outcome was the frequency and qualitative assessment of reporting of size, morphology, and location.

**Results:**

Of 3,074 articles, 262 articles were included. This comprised a total of 11,785 patients. Size was reported in 248 (95%) of the articles and was described with a measure for surface area in 83%, however, in 56%, definition of measurement is unknown. Intraclass coefficient (ICC) value for the reliability of size measurement was 0.94 for computed tomography (CT) scan and 0.87 for MRI scan. Morphology was reported in 172 (66%) of the articles and using a classification system in 23% of the studies. Location was reported in 220 (84%) of the studies.

**Conclusion:**

No consensus was found on the reporting of morphology, with non-validated classification systems and different terminologies used. For location, reporting in 9 zones is underreported. Size was well reported and measurements are more reliable for CT compared with MRI. As these prognostic factors guide clinical decision-making, we advocate the development of a standardized and validated OLT classification to reach uniform reporting in literature.

**Level of Evidence:**

Level III, systematic review.

## Introduction

Osteochondral lesions of the talus (OLTs) are lesions of the articular cartilage and the associated subchondral bone of the talar dome. The injuries have a high association with traumatic ankle injuries—such as lateral ankle sprains, ankle fractures, and syndesmotic injuries to the ankle.^1,2^

Considering the treatment of OLTs through either a conservative treatment protocol or a surgical intervention, there are several important factors deciding the type and invasiveness of the treatment. Understanding the wishes, needs, and quality of life of the patients with an OLT are considered one of the most important factors,^3,4^ and besides these factors, important radiological factors that are considered vital for the choice as well as the outcome of the treatment are lesion size, lesion location, and lesion morphology.^5,6^

First, size is a known predictor for clinical outcomes in different treatment strategies for OLTs, and especially concerning the outcomes of bone marrow stimulation (BMS).^7^ Furthermore, size is often described as a crucial metric to justify treatment decisions.^5^ However, different methodologies can be used to measure and calculate the size of a lesion, possibly obstructing the comparison between clinical results. Moreover, reliability of measurement for OLT size may differ when using either a computed tomography (CT) and/or an MRI scan.

Second, morphology can be considered an important factor in treatment decision-making.^5,8^ This is exemplified by the choice to perform a fixation procedure for OLTs as we know that solely as specific indication—namely, a fragmentous lesion with a specific size—are amenable to this technique.^9^ Consequently, it is important to strongly consider the lesion’s morphology toward deciding the most clinically suitable treatment strategy to yield the highest success rate possible for the individual patient.

Finally, for reporting of location, the talus can be divided into medial, central, and lateral zones.^10^ However, in 2007, Raikin *et al.*^11^ introduced a novel approach to geographically map the lesions in 9 zones. The majority of lesions are located in the posteromedial and centromedial zone.^10^ It has not been shown that the location of the OLT influences treatment outcome,^12^ however, it can be a basis for treatment decisions associated with for example an malleolar osteotomy, or anterior or posterior arthroscopy approach.^5,13^

Overall, size, location, and morphology influence clinical decision-making and appear prognostic for treatment outcome. Therefore, uniform, good quality reporting is crucial to appreciate the clinical outcome of studies. To reach standardization in reporting and work toward an evidence-based treatment decision tool, a clear overview of reporting in literature is vital. Therefore, the primary aim of this study was to evaluate the frequency and quality of reporting of size, morphology, and location of OLTs.

## Materials and Methods

**Figure 1.**
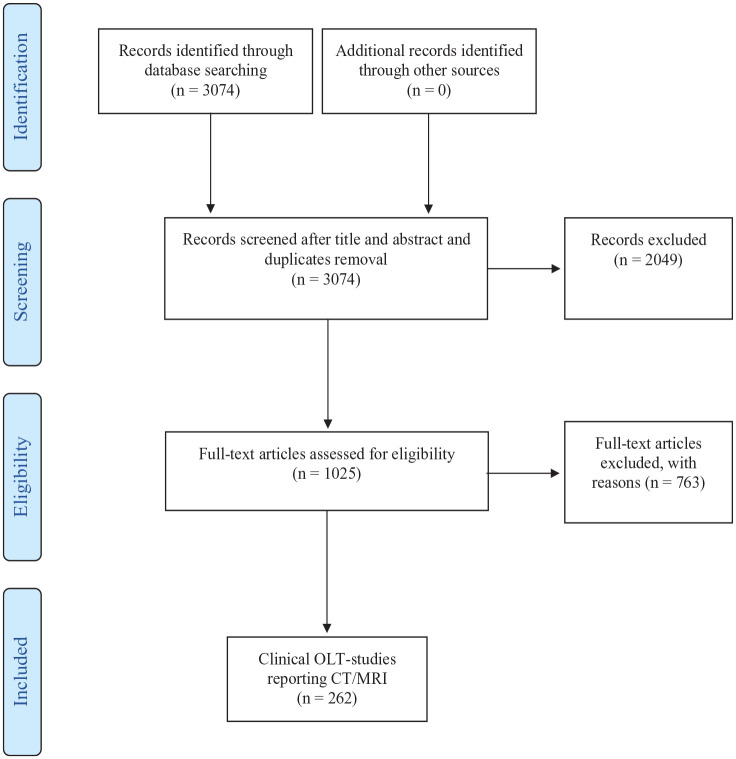
Selection of studies. OLT = osteochondral lesions of the talus; CT = computed tomography.

The present systematic review was prospectively registered at the PROSPERO register (**CRD42018081120**).^14^ Standardized guidelines from the Methodology of the Preferred Reporting Items for Systematic Reviews and Meta-Analyses (PRISMA) were followed, and are outlined in Figure 1.^15^

### Search Strategy

Articles were retrieved by digital searches in PubMed (MEDLINE), EMBASE (Ovid), and the Cochrane Library databases from 1996 until February 2023. The full search strategy is outlined in Appendix 1 in the Supplementary Material.

### Study Selection and Eligibility Criteria

Rayyan was used to independently screen all identified articles on title and abstract obtained through electronic search.^16^ Hereafter, full texts of potentially relevant articles were assessed on inclusion and exclusion criteria. The aim was to include all clinical studies, including treatment of at least 5 patients. The selection was performed by 2 reviewers (**PRvD** and **JD**). One reviewer, a medical doctor, with 6 years of clinical and research experience in the orthopedic surgery department and numerous publications, is joined by another reviewer, a medical student in their final year, with 4 years of engagement and a publication in orthopedic surgery department research. Discrepancies were discussed until consensus was reached. When no consensus could be reached, a third independent reviewer was consulted (**GK**) to reach a conclusion. Inclusion and exclusion criteria are detailed in Table 1.

**Table 1.** Inclusion and Exclusion Criteria.

Inclusion Criteria	Exclusion Criteria
1. Studies including at least 5 patients with OLTs 2. Primary and non-primary OLTs 3. Patients of all ages 4. All conservative and surgical treatment options	1. Treatment inappropriately described 2. Full-text article unavailable 3. Double publication, including overlap of patients 4. Duplicates 5. Cadaveric studies 6. Review studies 7. Conference abstracts 8. Not written in the English language 9. No reported CT/MRI pre-operative imaging

CT = computed tomography.

### Methodological Quality Assessment

The methodological quality of studies was assessed by 2 reviewers (**PRvD** and **JD**) using the methodological index for non-randomized studies (MINORS) tool.^17^ If both reviewers were inconclusive, a third independent reviewer was available and decisive. This instrument scored 8 items for non-comparative studies and 12 items for comparative studies. During the assessment, an article was scored a 0, 1, or 2 points. If an item was not reported, the item was scored 0. Items that were inadequately reported were scored “1” and adequately reported items were scored “2.” Thus, non-comparative studies can receive a maximum score of 16 points, whereas comparative studies can receive a maximum of 24 points.

### Data Extraction

Data of included studies were extracted (**PRvD**) using Microsoft Excel (version 2022, macOS Monterey). Standard study baseline characteristics and the following patient characteristics were retrieved: total number of articles, number of patients, sex, lesions, and ankles. In the case of categorical data, frequencies and percentages were calculated. In the case of continuous data, weighted averages were calculated with the associated ranges of these averages. To compare the quality of size measurement, intraclass coefficients (ICCs) regarding size measurement reliability were collected. This review did not focus on radiographs as pre-operative imaging as the sensitivity and specificity are inferior to CT and MRI, and therefore, radiographs are generally not solely part of standard practice anymore.^18^ In addition, this review did not focus on arthroscopic measurement as that is not a pre-operative treatment decision tool.

### Statistical and Data Analysis

#### Size reporting

The primary outcome measure for size reporting was defined as the following outcome: percentage of studies reporting any measure of size. Whenever size was reported in the specific literature, the following secondary outcomes were assessed: percentage of studies reporting size measurement through diameter (anteroposterior [AP], mediolateral [ML]), depth, surface (mm^2^), and as volume (mm^3^). In addition, reliability of size measurement of pre-operative CT and MRI was averaged per modality and the definitions that were used to calculate the surface and volume were analyzed.

#### Morphology reporting

The outcome measure for reporting of lesion morphology was the percentage of studies reporting any kind of morphology. Consequently, whenever lesion morphology was reported, the manner of morphological description was also analyzed (either through the reported description with a CT/MRI classification system or through a self-reported qualitative method). Other outcomes in this group are the reporting distribution of the qualitatively described morphology in one of the (major) 3 OLT morphology groups: cystic, fragment, or crater.

#### Location reporting

The outcome measure for the location reporting was defined as the following outcome: percentage of studies reporting of location. Whenever location was reported in the specific literature, the following manners of reporting were analyzed concerning frequency of reporting: percentage of location measurement reported as either (1) medial, central, lateral, (2) shoulder versus non-shoulder localization, or as (3) 9-zone grid system localization system.

## Results

### Search Results, Screening, and Selection of Studies

The literature search resulted in 3,074 articles. After title and abstract screening in Rayyan, 2,049 articles were excluded based on the inclusion and exclusion criteria.^16^ The remaining 1,025 studies remained for full-text screening, of which 763 articles were excluded. Finally, after the full-text screening and excluding studies missing CT/MRI reporting, 262 studies were included. There was consensus in all cases of article selection between the first 2 authors. The PRISMA flowchart is detailed in Figure 1.

### Evaluation of Characteristics of Included Studies

Included clinical studies reported a total of 11,785 patients in 11,862 ankles. The average age of patients in the included papers was 36 (range, 11-68) years. Most patients were male (59%). The included clinical studies mostly covered bone marrow and/or cartilage stimulation, matrix-assisted BMS, osteo(chondral) transplantation techniques, and cartilage implantation techniques. Patient demographics and reported details of variables are summarized in Tables 2 and 3.

**Table 2.** Patient Characteristics.

Variables	Clinical OLT Studies That Used CT and/or MRI (*n* = 262)
Male/female (%)	59/41
Median age in years (range)	36 (11-68)
No. of patients	11,785
No. of ankles	11,862

OLT = osteochondral lesions of the talus; CT = computed tomography; MRI = magnetic resonance imaging.

**Table 3.** Reporting of Key Variables.

	Total No. of Studies (*n*)	Studies That Report Variable (*n*)	%
Size reporting			
Clinical studies that use CT/MRI and report size	262	248	95
Clinical studies that use CT/MRI and report reliability measurement of size	262	7	3
Morphology reporting			
Clinical studies that use CT/MRI: and report any morphology description	262	172	66
Reported classification systems	262	61	23
When used:			
Hepple *et al.*			
MRI	61	29	47
Anderson *et al.*			
MRI	61	16	25
Giannini’s *et al.*			
MRI	61	11	18
Ferkel *et al.*			
CT	61	1	2
Dipaola *et al.*			
MRI	61	1	2
Nelson *et al.*			
MRI	61	1	2
Mintz *et al.*			
MRI	61	1	2
Taranow *et al.*			
MRI	61	1	2
Free morphology descriptions	262	111	42
When used:			
Cystic	111	39	37
Contained	111	4	3
Subchondral cyst	111	35	34
Fragment	111	8	8
Loose bodies	111	3	3
Partly or completely displaced	111	3	3
Unstable	111	1	1
(Partial) fragmentation	111	1	1
Crater	111	4	4
Subchondral plate irregularities	111	2	2
Compression	111	1	1
Some collapse	111	1	1
Explicitly specified as inclusion criteria	111	58	53
Cystic vs. non-cystic	111	43	38
Contained vs. uncontained	111	8	7
Osteochondral defect vs. degenerative chondral defect	111	2	2
Chondral vs. osteochondral	111	1	1
Acute vs. chronic	111	1	1
Stable vs. unstable	111	1	1
Other descriptions	111	2	2
Degenerative chondral lesion	111	2	2
Location reporting			
Medial/central/lateral that used CT/MRI	262	220	84
9-zone grid that used CT/MRI	262	83	32
Corrected for publication year after introduction of 9-zone grid in 2007	244	83	34
Shoulder vs. non-shoulder	262	8	3

CT = computed tomography; MRI = magnetic resonance imaging.

### Methodological Quality

For all cases, there was full agreement between the first 2 authors on the assessment of methodological quality of the studies. After MINORS assessment, 205 studies were non-comparative and 57 were comparative studies. Non-comparative studies scored an average of 10 points (2-14) where a maximum score of 16 was possible. Comparative studies scored an average of 18 points (12-22), where a maximum score of 24 was possible. Finally, 68 papers reported prospective and 143 papers reported retrospective collection of data. Detailed information regarding MINORS scores is included in Appendix 2.

### Size Reporting

The primary outcome measure for size reporting resulted in 95% of clinical papers that used CT or MRI (248/262 papers). Measurements were conducted on CT in 32 studies, whereas 189 studies reported size for MRI. Overall, the size was most frequently reported as a measure of surface area (83%). The second most frequently reported dimension was the AP diameter reported with a reporting rate of 23%. Overall, size was relatively most reported in the CT only group. Volume was least reported, consisting of 22% for CT and 10% for MRI. Details per dimension and modality are reported in Table 4.

**Table 4.** Size Measurement Reporting.

Measurement Variables	Combined (*n* = 262)		CT (*n* = 32)		MRI (*n* = 189)		MRI and/or CT (*n* = 27)	
	Count	%	Count	%	Count	%	Count	%
Anteroposterior (mm)	61	23	19	59	32	17	8	20
Mediolateral (mm)	46	18	19	59	20	11	6	15
Depth (mm)	57	22	17	53	29	15	6	15
Surface (mm^2^)	218	83	24	75	164	87	17	40
Volume (mm^3^)	31	12	7	22	17	9	4	10

CT = computed tomography; MRI = magnetic resonance imaging; mm = millimeter; % = percentage.

Multiple formulas were used to calculate both the surface and size. However, in 147 studies (56%), there was no definition provided (Table 5). The most reported definition is the ellipsoid formula, which has been proposed by Choi *et al.*^19,20^

**Table 5.** Reporting of Surface/Volume Definitions.

Definition	Studies Reporting Size (*n* = 262)	
	Count	%
Unknown/not reported	144	56
Ellipsoid formula	61	23
Dimensions (i.e., AP/ML [/Depth])	50	19
Segmentation	2	1
Circle formula	1	0
Diameter (i.e., one dimension)	1	0

Reliability of size measurement for CT and or MRI was reported for 6 studies.^21-26^ A weighted average ICC of 0.94 was found for CT, compared with 0.87 for MRI (Table 6).

**Table 6.** Intraclass Coefficient Reporting and Measurement for CT and MRI.

Literature	ICC in CT			ICC in MRI		
Becher *et al.*^21^	n.a.			0.94		
Li *et al.*^22^	n.a.			0.76		
Ahn *et al.*^24^	n.a.			0.83		
Seo *et al.*^26^	0.90-0.93			0.90-0.93		
Reilingh *et al.*^23^	0.98			n.a.		
	AP	ML	Depth	AP	ML	Depth
Deng *et al.*^25^	0.98	0.97	0.99	0.98	0.96	0.98
Rikken *et al.*^5^	0.88	0.91	0.82			

CT = computed tomography; MRI = magnetic resonance imaging; ICC = intraclass coefficient; n.a. = not applicable; AP = anteroposterior; ML = mediolateral.

### Morphology Reporting

A description of OLT morphology was reported in 66% (*n* = 172/262) of clinical articles that used CT or MRI. For 23% (61/262), morphology was reported in a classification system. If a classification system was used, the system introduced by Hepple *et al.*,^27^ Anderson *et al.*,^28^ and Giannini *et al.*^29^ were dominantly used. Morphology was most frequently reported in a free description in 42% of the studies (111/262). Apart from classification system usage, descriptions of OLT morphology could be classified in several groups: (1) cystic, (2) fragment, (3) crater, (4) specific inclusion criteria where 2 morphology groups are compared, and (5) other categories. Results of all groups are shown in Table 3.

### Location Reporting

Location was reported in 220 studies (84%) as medial, central, and lateral in literature. According to the 9-zone grid of Raikin *et al.*,^11^ location distribution data were reported in 32% of the studies (Table 3).

### Paradox in Reporting

**Figure 2.**
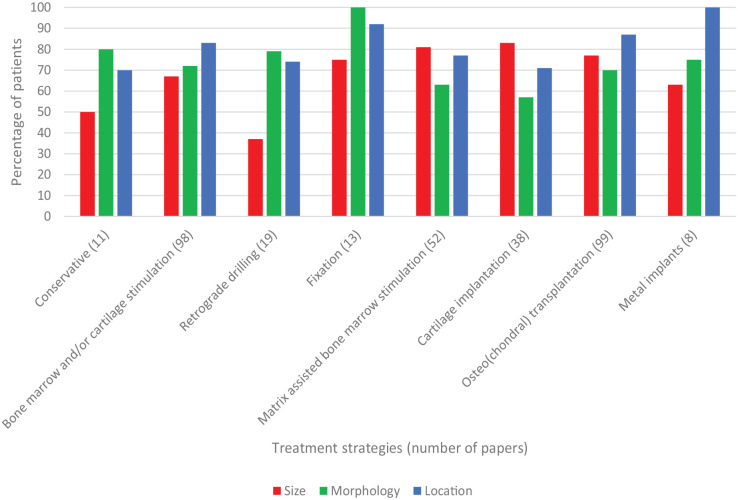
Reporting per treatment strategy (*n* = 338).

In 95% of cases, size was reported (248/262). Papers providing individual data on size, morphology, location, and treatment strategy were included in Figure 2. Paradoxically, within this data set, size was less frequently reported (< 95%) due to the exclusion of papers without individual treatment strategy data. Several papers reported individual data on multiple treatment strategies resulting in 338 reported treatment strategies in 262 papers. Detailed treatment reporting of size, morphology, and location reporting is specified in Figure 2.

## Discussion

In the literature on the treatment of OLTs, a majority of the studies reports size (95%), morphology (66%), and location (84%), if CT imaging and MRI are used. Size was primarily reported as surface area (83%), and measured on MRI (72%), while volume was reported the least (12%). In 56% of the studies, it was unclear how the authors calculate the surface and volume. No consensus was found on the way of reporting the morphology of the OLT. Morphology was most frequently reported in a free descriptive manner in most studies (42%). One CT classification and 7 classifications for MRI were reported. Location was most frequently reported in a medial/central/lateral manner (84%), including a more detailed 9-zone grid (32%). We advocate the standard usage of a reporting protocol when describing scientific clinical work on osteochondral lesions of the ankle.

### Size

Size has found to be an important prognostic factor for outcome after surgical treatment.^7^ In recent years, Choi *et al.*^19^ reported for lesions larger than 150 mm^2^ a high failure rate (80%) in patients undergoing BMS. In addition to this study, Flynn *et al.*^30^ concluded there should be inclusion criteria for size before a replacement strategy should be used to reach better results after surgery. Size measurements can be considered reliable for both CT assessments and MRI assessments in literature.^25^ It is important to take lesion characteristics into account regarding size measurement. D’Ambrosi *et al.*^31^ described that lesions were bigger sized in both CT and MRI if bone marrow edema was present. Our meta-analysis demonstrates that size appears to be more reliably evaluated on CT than on MRI, which seems relevant for clinical practice. However, a future larger study will have to prove this. Size is well reported in literature with 95%.

### Morphology

The variety of reporting throughout the literature showed that there is no clear consensus on how to report morphology. A minority of the studies used 1 of 8 identified classification systems. In addition to these classification systems, many other descriptions were presented in literature, including dichotomous variables, such as cystic versus non-cystic or contained versus uncontained.^32,33^ In addition, making a clear distinction between a fragmentous or crater-like lesion can be burdensome. This due to the intermediate appearance of some lesions, making it challenging to systematically categorize lesion morphology. Since the overview of O’Loughlin *et al.*,^8^ multiple new classification systems have been developed.^28,29,34^ Contradictory to the 9-zone grid, none of the new classification systems have been validated.^35^ It is essential to have a uniform and increased (66% currently) reporting of morphology in OLT studies.

### Interaction Between Size and Morphology

Measurement of size can be performed in different ways but is often not reported (56% of studies). When reported, multiple methods are used. This results in a lack of uniformity in the literature, making it less appropriate to compare between different studies.

**Figure 3.**
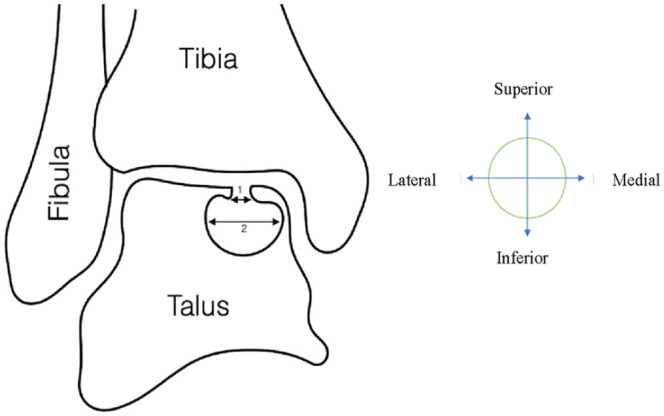
Subchondral cyst and size measurement.

**Figure 4.**
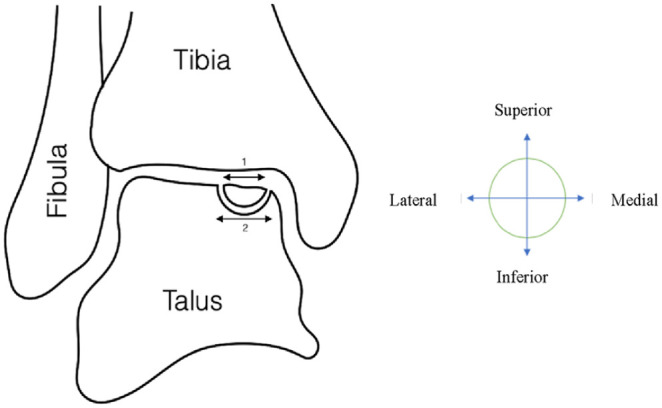
Fragment and size measurement.

Visually explained, a subchondral cyst (Figure 3) can be measured in different manners. This type of lesion can be measured (number 2, Figure 3) in the cyst (i.e., greatest dimension) compared with the size measured on the articular surface in the joint (number 1, Figure 3). Therefore, it makes much difference where the lesion size is measured. When the OLT contains a fragment, size measurement variation seems less obvious (Figure 4). The surface area (number 1, Figure 4) can be slightly overestimated compared with the fragment size (number 2, Figure 4). In addition, one could argue to either include or exclude the surrounding sclerotic zone. Therefore, utilizing the articular surface area as most frequently reported in literature is debatable. OLTs suitable for BMS therapy (Figure 3) have different morphological properties than lesions containing a large fragment suitable for fixation (Figure 4). It may be suggested to measure OLT size in a volumetric 3-dimensional combined morphological manner to increase standardization of size measurement.

### Location

OLTs’ location is historically most frequently reported in the anteromedial and posteromedial zones of the talus.^36^ In 2007, Raikin *et al.*^11^ invented a novel way of reported OLTs location. Location reporting proceeded in 3 rows (anterior, medial, and posterior) and 3 columns (medial, central, and lateral). A recent systematic review shows that location was reported for 2087 patients according to the 9-zone grid.^10^ The most frequently reported zones consisted of the posteromedial and centromedial zone for 59% percent of lesions.^10^ In the current study, lesions were reported in 32% of all included studies and in 84% of those, reported according to the historic medial, central, and lateral layouts compared with 32% of lesions reported according to the 9-zone grid. This underreporting persists even after correcting for publication year, with 244 (93%) of the included papers being from 2007 or later results in 34% location reporting according to the 9-zone grid. In contrast, OLTs location is relevant in pre-operative planning.^12^ Therefore, future research should report OLTs location in a standardized manner according to the 9-zone grid before solid conclusions could be drawn regarding treatment and outcome in combination with size and morphology.

### Recommendations

In this study, designed to get insight in reporting of size, morphology, and location, we found that size is frequently reported. A current problem is a lack of reporting how surface and volume measurements were made and calculated. In addition, no consensus was found on the reporting of morphology. A clinically relevant solution would be a uniform reporting of size, morphology, and location. Size in a 2- or 3-dimensional manner, morphology in a validated classification, and location in the validated 9-zone grid. A more consistent reporting of these variables in future studies has the potential to facilitate a deeper understanding of the relationship between size, morphology and location, treatment approaches, and patient outcomes. This could improve treatment of osteochondral lesions in the future, as this deeper understanding can be used for an evidence-based treatment tool. In the meantime, we recommend to report at least the size in 3 dimensions, morphology categorized as cystic, fragmentous, crater, or other, and location in the 9-zone grid.

### Limitations and Strengths

This review did not focus on arthroscopic measurement as that is not a pre-operatively treatment decision tool in clinical practice. In addition, the reliability of size measurement seems more favorable to CT but should be taken with caution as only one study, Deng *et al.*,^25^ provided a comparative statistical tests. The second limitation of this study is that most of the studies were retrospective and thus of low methodological quality. Prospective studies are usually better structured and therefore better quality reporting can be expected, as *a priori* variables of interest are defined. This study’s third limitation is that it only considered clinical studies that incorporated CT/MRI reporting, which was done to align with current practice. A major strength of our systematic review is a large number of included clinical studies and thus an overview of almost the entire literature. Another strength is that this study included all prominent, seemingly relevant factors influencing treatment decisions for clinicians in daily practice. Finally, this study was executed systematically, adhering to the PRISMA guidelines, and using a validated tool for quality assessment (MINORS).

## Conclusion

Size was reported in 95% of the studies. However, in 56% of the studies, it was not reported how surface and volume measurements were made and calculated, decreasing inter-study comparability. Higher ICC values were reported for CT assessments compared with MRI assessments. No consensus was found on the reporting of morphology (reported in 66% of studies), with a minority of the authors using non-validated classification systems, in an addition to a variety of dichotomous descriptions. Location (was only in a minority of the cases reported in a 9-zone grid (32% of studies that reported location). Uniformity in reporting is important for clinical decision-making and can impact treatment outcomes. A validated and standardized OLT classification system including all factors (size, morphology, and location) is necessary to reach uniform reporting in literature.

## Supplemental Material

sj-docx-1-car-10.1177_19476035241229026 – Supplemental material for Reporting of Morphology, Location, and Size in the Treatment of Osteochondral Lesions of the Talus in 11,785 Patients: A Systematic Review and Meta-Analysis Supplemental material, sj-docx-1-car-10.1177_19476035241229026 for Reporting of Morphology, Location, and Size in the Treatment of Osteochondral Lesions of the Talus in 11,785 Patients: A Systematic Review and Meta-Analysis by Pascal R. van Diepen, Frank F. Smithuis, Julian J. Hollander, Jari Dahmen, Kaj S. Emanuel, Sjoerd A.S. Stufkens and Gino M.M.J. Kerkhoffs in CARTILAGE
