# Supplementary material for: Reporting of Morphology, Location, and Size in the Treatment of Osteochondral Lesions of the Talus in 11,785 Patients: A Systematic Review and Meta-Analysis
Source: Cartilage. 2024 Feb 16;17(1):18–27. doi: 10.1177/19476035241229026 (PMC11569679; doi:10.1177/19476035241229026)
Supplement: sj-docx-1-car-10.1177_19476035241229026 – Supplemental material for Reporting of Morphology, Location, and Size in the Treatment of Osteochondral Lesions of the Talus in 11,785 Patients: A Systematic Review and Meta-Analysis [file sj-docx-1-car-10.1177_19476035241229026.docx]

# **APPENDIX 1 SEARCH**

**Search strategy until February 1^th^, 2023**

| **PUBMED** | **Search terms** |
| --- | --- |
| #1 | "Osteochondritis Dissecans"[Mesh] |
| [#](https://www.ncbi.nlm.nih.gov/pubmed/advanced)2 | Osteochondritis dissecans[tiab] OR osteochondrosis dissecans[tiab] OR osteochondrolysis[tiab] OR OCD[tiab] OR OLT[tiab] |
| #3 | (osteochondral[tiab] OR chondral[tiab] OR transchondral[tiab] OR cartilage*[tiab]) AND (defect*[tiab] OR lesion*[tiab]) |
| #4 | #1 OR #2 OR #3 |
| #5 | "Talus"[Mesh] |
| #6 | talus[tiab] OR talar*[tiab] OR ankle[tiab] |
| #7 | #5 OR #6 |
| #8 | #4 AND #7 |
|  |  |
| **EMBASE** | **Search terms** |
| #1 | (osteochondritis dissecans/or (osteochondritis dissecans or osteochondrosis dissecans or osteochondrolysis or OCD or OLT).ti,ab,kw. or ((osteochondral or chondral or osteochondral or transchondral or cartilage*) adj3 (defect* or lesion*)).ti,ab,kw.) and (talus/ or (talus or talar* or ankle).ti,ab,kw.) |
|  |  |
| **COCHRANE** | **Search terms** |
| #1 | MeSH descriptor: [Osteochondritis Dissecans] explode all trees |
| #2 | osteochondritis dissecans or osteochondrosis dissecans or osteochondrolysis or OCD or OLT:ti,ab,kw (Word variations have been searched) |
| #3 | (osteochondral or chondral or transchondral or cartilage*) and (defect* or lesion*):ti,ab,kw (Word variations have been searched) |
| #4 | #1 or #2 or #3 |
| #5 | MeSH descriptor: [Talus] explode all trees |
| #6 | Talus or talar* or ankle*:ti,ab,kw (Word variations have been searched) |
| #7 | #5 or #6 |

# **APPENDIX 2 MINORS**

|  | | | | | | | | | | | | | |
| --- | --- | --- | --- | --- | --- | --- | --- | --- | --- | --- | --- | --- | --- |
| MINORS | | | | | | | | | Additional criteria for comparative studies | | | |  |
| Study | A clearly stated aim | Inclusion of consecutive patients | Prospective collection of data | Endpoint appropiate to the aim of the study | Unbiased assesment of the study endpoint | Follow up period appropiate to the aim of the study | Lost of follow up less than 5% | Prospective calculation of study size | An adequate control group | Contampory group | Baseline equivalent of groups | Adequate statistical analysis | Total |
| Fayyad, 2017 | 2 | 2 | 2 | 2 | 2 | 2 | 0 | 0 | - | - | - | - | 11/16 |
| Adams, 2011 | 2 | 1 | 1 | 2 | 2 | 2 | 2 | 0 | - | - | - | - | 11/16 |
| Adams ,2018 | 2 | 2 | 2 | 2 | 0 | 2 | 2 | 1 | 1 | 2 | 1 | 0 | 17/24 |
| Ahmad, 2016 | 2 | 1 | 2 | 2 | 2 | 1 | 2 | 0 | 2 | 2 | 2 | 0 | 16/24 |
| Ahmad, 2017 | 2 | 2 | 2 | 2 | 2 | 1 | 2 | 0 | - | - | - | - | 13/16 |
| Anders, 2011 | 2 | 2 | 0 | 2 | 2 | 1 | 2 | 0 | - | - | - | - | 11/16 |
| Angthong, 2013 | 2 | 1 | 2 | 2 | 2 | 0 | 2 | 0 | - | - | - | - | 11/16 |
| Trattnig, 2012 | 1 | 2 | 0 | 2 | 2 | 2 | 2 | 0 | 2 | 2 | 2 | 1 | 18/24 |
| Manuela, 2017 | 1 | 2 | 1 | 2 | 2 | 1 | 2 | 0 | - | - | - | - | 10/16 |
| Assenmacher, 2001 | 2 | 0 | 1 | 2 | 2 | 1 | 1 | 0 | - | - | - | - | 8/16 |
| Battaglia, 2011 | 2 | 0 | 0 | 2 | 2 | 2 | 1 | 0 | - | - | - | - | 10/16 |
| Battaglia, 2011 | 2 | 0 | 0 | 2 | 2 | 2 | 2 | 0 | - | - | - | - | 11/16 |
| Becher, 2010 | 1 | 0 | 2 | 2 | 2 | 2 | 1 | 0 | - | - | - | - | 10/16 |
| Becher, 2018 | 2 | 0 | 1 | 1 | 2 | 2 | 1 | 0 | 1 | 2 | 2 | 1 | 14/24 |
| Becher, 2005 | 2 | 2 | 2 | 2 | 2 | 2 | 2 | 0 | - | - | - | - | 14/16 |
| Becher, 2015 | 2 | 1 | 0 | 2 | 1 | 2 | 2 | 0 | - | - | - | - | 10/16 |
| Beck, 2016 | 2 | 1 | 2 | 2 | 1 | 2 | 1 | 0 | - | - | - | - | 11/16 |
| Berlet, 2011 | 2 | 1 | 2 | 2 | 2 | 2 | 0 | 0 | - | - | - | - | 11/16 |
| Bleazey, 2012 | 2 | 1 | 1 | 2 | 1 | 2 | 2 | 0 | - | - | - | - | 10/16 |
| Buda, 2015 | 2 | 1 | 1 | 2 | 2 | 2 | 2 | 0 | 1 | 2 | 2 | 2 | 18/24 |
| Buda, 2013 | 2 | 0 | 2 | 2 | 2 | 2 | 2 | 0 | - | - | - | - | 12/16 |
| Cadossi, 2014 | 2 | 0 | 2 | 2 | 2 | 2 | 2 | 0 | 2 | 2 | 2 | 2 | 20/24 |
| Giannini, 2013 | 2 | 2 | 2 | 2 | 0 | 2 | 0 | 0 | - | - | - | - | 10/16 |
| Volpi, 2014 | 2 | 0 | 0 | 2 | 1 | 2 | 2 | 0 | - | - | - | - | 10/16 |
| Chen, 2015 | 2 | 0 | 2 | 2 | 2 | 2 | 1 | 0 | - | - | - | - | 11/16 |
| Choi, 2013 | 2 | 1 | 1 | 2 | 2 | 1 | 2 | 0 | 2 | 1 | 1 | 2 | 18/24 |
| Choi, 2016 | 2 | 0 | 1 | 2 | 2 | 1 | 2 | 0 | 2 | 2 | 2 | 2 | 17/24 |
| Choi, 2013 | 2 | 2 | 1 | 2 | 2 | 1 | 2 | 0 | - | - | - | - | 11/16 |
| Choi, 2009 | 2 | 1 | 1 | 1 | 2 | 2 | 2 | 0 | - | - | - | - | 10/16 |
| Cuttica, 2011 | 1 | 0 | 1 | 2 | 1 | 2 | 2 | 0 | - | - | - | - | 8/16 |
| D’Ambrosi, 2017 | 2 | 2 | 1 | 2 | 2 | 2 | 2 | 0 | - | - | - | - | 12/16 |
| D’Ambrosi, 2018 | 2 | 1 | 1 | 2 | 2 | 2 | 2 | 0 | 2 | 2 | 0 | 2 | 18/16 |
| D’Ambrosi, 2017 | 2 | 1 | 1 | 2 | 2 | 2 | 2 | 0 | 0 | 1 | 0 | 2 | 14/24 |
| D’Ambrosi, 2018 | 2 | 2 | 0 | 2 | 2 | 2 | 2 | 0 | - | - | - | - | 12/16 |
| D’Ambrosi, 2017 | 2 | 1 | 1 | 2 | 0 | 2 | 1 | 2 | - | - | - | - | 12/16 |
| Dekker, 2018 | 2 | 2 | 1 | 2 | 0 | 2 | 0 | 0 | - | - | - | - | 8/16 |
| DeSandis, 2018 | 1 | 2 | 1 | 2 | 0 | 2 | 0 | 0 | 2 | 2 | 2 | 2 | 16/24 |
| Domayer, 2012 | 2 | 1 | 0 | 2 | 2 | 2 | 2 | 0 | 1 | 2 | 2 | 2 | 18/24 |
| Domayer, 2011 | 2 | 2 | 0 | 2 | 0 | 1 | 0 | 0 | - | - | - | - | 7/16 |
| Dunlop, 2013 | 1 | 2 | 1 | 2 | 2 | 2 | 2 | 0 | - | - | - | - | 11/16 |
| El-Rashidy, 2011 | 2 | 0 | 1 | 2 | 2 | 1 | 2 | 0 | - | - | - | - | 9/16 |
| Emre, 2012 | 2 | 0 | 2 | 2 | 2 | 1 | 2 | 0 | - | - | - | - | 11/16 |
| Nam, 2009 | 2 | 0 | 2 | 2 | 2 | 2 | 2 | 0 | - | - | - | - | 12/16 |
| Flynn, 2016 | 2 | 2 | 1 | 2 | 2 | 2 | 2 | 0 | - | - | - | - | 12/16 |
| Fraser, 2016 | 2 | 2 | 1 | 2 | 2 | 2 | 2 | 0 | - | - | - | - | 13/16 |
| Görmeli, 2015 | 2 | 2 | 2 | 2 | 2 | 2 | 2 | 0 | 2 | 2 | 2 | 2 | 22/16 |
| Gao, 2017 | 2 | 1 | 1 | 2 | 2 | 2 | 2 | 0 | 2 | 2 | 1 | 0 | 16/24 |
| Gianakos, 2015 | 2 | 1 | 1 | 1 | 2 | 2 | 2 | 0 | - | - | - | - | 10/16 |
| Giannini, 2010 | 2 | 0 | 0 | 2 | 0 | 2 | 0 | 0 | - | - | - | - | 6/16 |
| Gobbi, 2006 | 2 | 0 | 0 | 2 | 2 | 2 | 2 | 0 | 2 | 2 | 0 | 1 | 15/24 |
| Gottschalk, 2017 | 2 | 2 | 2 | 2 | 0 | 2 | 0 | 0 | - | - | - | - | 1/16 |
| Gu, 2017 | 2 | 1 | 2 | 2 | 0 | 1 | 1 | 0 | - | - | - | - | 9/16 |
| Haene, 2012 | 1 | 0 | 2 | 1 | 2 | 2 | 2 | 0 | - | - | - | - | 10/16 |
| Haleem, 2012 | 2 | 2 | 1 | 2 | 0 | 1 | 2 | 1 | 1 | 2 | 0 | 0 | 14/24 |
| Han, 2006 | 2 | 0 | 2 | 2 | 2 | 2 | 2 | 0 | 1 | 2 | 2 | 0 | 17/24 |
| Hangody, 1997 | 1 | 0 | 1 | 1 | 2 | 1 | 1 | 0 | - | - | - | - | 7/16 |
| Hangody, 2001 | 2 | 2 | 0 | 2 | 2 | 2 | 2 | 0 | - | - | - | - | 12/16 |
| Hannon, 2016 | 2 | 2 | 1 | 2 | 2 | 1 | 2 | 0 | 2 | 2 | 2 | 2 | 20/24 |
| Hintermann, 2015 | 2 | 2 | 2 | 2 | 0 | 2 | 0 | 0 | - | - | - | - | 10/16 |
| Nehrer, 2011 | 1 | 2 | 2 | 2 | 0 | 2 | 0 | 0 | - | - | - | - | 5/16 |
| Hu, 2013 | 2 | 2 | 0 | 2 | 0 | 2 | 0 | 0 | - | - | - | - | 8/16 |
| Ikoma, 2019 | 2 | 1 | 2 | 2 | 2 | 2 | 2 | 0 | - | - | - | - | 13/16 |
| Jancewicz, 2004 | 2 | 0 | 0 | 2 | 2 | 0 | 1 | 0 | - | - | - | - | 9/16 |
| Jung, 2018 | 2 | 1 | 0 | 2 | 2 | 1 | 2 | 0 | - | - | - | - | 10/16 |
| Kanatli, 2017 | 2 | 0 | 1 | 2 | 2 | 2 | 2 | 0 | - | - | - | - | 10/16 |
| Kennedy, 2011 | 2 | 1 | 1 | 2 | 2 | 2 | 1 | 0 | - | - | - | - | 12/16 |
| Kerkhoffs, 2016 | 2 | 1 | 2 | 2 | 2 | 0 | 2 | 0 | - | - | - | - | 11/16 |
| Kim, 2013 | 2 | 2 | 1 | 2 | 0 | 2 | 0 | 0 | - | - | - | - | 8/16 |
| Kim, 2012 | 2 | 2 | 2 | 2 | 2 | 2 | 2 | 0 | - | - | - | - | 14/16 |
| Klammer, 2015 | 2 | 0 | 1 | 2 | 2 | 2 | 2 | 0 | - | - | - | - | 10/16 |
| Kokkinakis, 2008 | 1 | 0 | 1 | 2 | 1 | 2 | 2 | 0 | - | - | - | - | 8/16 |
| Kolker, 2004 | 2 | 0 | 1 | 2 | 2 | 2 | 2 | 0 | - | - | - | - | 10/16 |
| Koulalis, 2002 | 1 | 0 | 0 | 2 | 1 | 1 | 2 | 0 | - | - | - | - | 7/16 |
| Koulalis, 2004 | 2 | 1 | 1 | 2 | 2 | 0 | 2 | 0 | - | - | - | - | 10/16 |
| Kramer, 2015 | 2 | 2 | 1 | 2 | 0 | 2 | 0 | 0 | - | - | - | - | 8/16 |
| Kreuz, 2008 | 1 | 0 | 2 | 2 | 2 | 2 | 2 | 0 | - | - | - | - | 11/16 |
| Kreuz, 2006 | 2 | 0 | 2 | 2 | 2 | 2 | 2 | 0 | - | - | - | - | 12/16 |
| Kubosch, 2016 | 2 | 0 | 0 | 2 | 2 | 2 | 2 | 0 | - | - | - | - | 10/16 |
| Kumai, 2002 | 0 | 0 | 0 | 2 | 0 | 2 | 0 | 0 | - | - | - | - | 4/16 |
| Kuni, 2012 | 1 | 0 | 1 | 2 | 1 | 1 | 0 | 0 | - | - | - | - | 5/16 |
| Kwak, 2014 | 2 | 2 | 2 | 2 | 2 | 1 | 2 | 0 | - | - | - | - | 13/16 |
| Lee, 2015 | 2 | 0 | 2 | 2 | 2 | 2 | 2 | 0 | 1 | 2 | 2 | 2 | 19/24 |
| Lee, 2009 | 2 | 0 | 0 | 2 | 2 | 0 | 2 | 0 | - | - | - | - | 8/16 |
| Tailee, 2013 | 2 | 0 | 2 | 2 | 2 | 1 | 2 | 0 | - | - | - | - | 11/16 |
| Lee, 2015 | 2 | 0 | 1 | 2 | 1 | 2 | 2 | 0 | 2 | 2 | 2 | 2 | 17/24 |
| Pagliazzi, 2016 | 1 | 2 | 1 | 2 | 0 | 2 | 0 | 0 | - | - | - | - | 7/16 |
| Li, 2018 | 1 | 2 | 0 | 1 | 1 | 2 | 0 | 0 | 2 | 2 | 2 | 2 | 15/24 |
| Li, 2017 | 2 | 2 | 2 | 2 | 2 | 2 | 1 | 0 | - | - | - | - | 13/16 |
| Lin, 2010 | 1 | 2 | 1 | 2 | 2 | 0 | 2 | 0 | - | - | - | - | 9/16 |
| Link, 2006 | 0 | 0 | 1 | 2 | 1 | 2 | 2 | 0 | - | - | - | - | 7/16 |
| Magnan, 2012 | 0 | 2 | 0 | 2 | 0 | 2 | 0 | 0 | - | - | - | - | 6/16 |
| Mei-Dan, 2012 | 2 | 2 | 2 | 2 | 2 | 2 | 2 | 0 | 2 | 2 | 2 | 2 | 22/16 |
| Richer, 2017 | 2 | 2 | 2 | 2 | 1 | 2 | 1 | 0 | - | - | - | - | 12/16 |
| Nakasa, 2018 | 2 | 0 | 0 | 2 | 2 | 0 | 1 | 0 | 1 | 2 | 2 | 0 | 12/24 |
| Nakasa, 2019 | 2 | 0 | 1 | 2 | 1 | 0 | 2 | 0 | - | - | - | - | 7/16 |
| Nakasa, 2018 | 2 | 0 | 1 | 2 | 2 | 0 | 2 | 0 | - | - | - | - | 8/16 |
| Orr, 2017 | 2 | 1 | 2 | 2 | 2 | 2 | 2 | 0 | - | - | - | - | 13/16 |
| Park, 2015 | 1 | 0 | 2 | 2 | 2 | 2 | 2 | 0 | - | - | - | - | 11/16 |
| Park, 2018 | 2 | 0 | 1 | 1 | 2 | 2 | 2 | 0 | 2 | 2 | 2 | 2 | 17/24 |
| Petersen, 2014 | 2 | 2 | 2 | 2 | 0 | 1 | 0 | 0 | - | - | - | - | 9/16 |
| Raikin, 2004 | 2 | 0 | 0 | 1 | 0 | 0 | 1 | 0 | - | - | - | - | 4/16 |
| Raikin, 2007 | 2 | 2 | 1 | 2 | 2 | 2 | 2 | 0 | - | - | - | - | 13/16 |
| Reilingh, 2014 | 2 | 2 | 1 | 2 | 0 | 2 | 1 | 0 | - | - | - | - | 9/16 |
| Reilingh, 2018 | 2 | 0 | 2 | 2 | 2 | 0 | 2 | 0 | 1 | 2 | 1 | 2 | 16/24 |
| Reilingh, 2016 | 2 | 0 | 0 | 2 | 2 | 0 | 2 | 0 | - | - | - | - | 8/16 |
| Reilingh, 2016 | 2 | 2 | 2 | 2 | 2 | 1 | 0 | 1 | 2 | 2 | 2 | 0 | 18/24 |
| Richter, 2013 | 2 | 2 | 2 | 2 | 2 | 2 | 2 | 0 | - | - | - | - | 14/16 |
| Ronga, 2005 | 0 | 0 | 0 | 2 | 1 | 1 | 2 | 0 | - | - | - | - | 6/16 |
| Ross, 2016 | 2 | 0 | 1 | 2 | 2 | 1 | 2 | 0 | - | - | - | - | 9/16 |
| Rossbach, 2016 | 2 | 0 | 1 | 2 | 1 | 0 | 2 | 0 | - | - | - | - | 7/16 |
| Sadlika, 2017 | 2 | 2 | 2 | 2 | 0 | 2 | 0 | 0 | 2 | 2 | 2 | 2 | 18/24 |
| Sadlika, 2018 | 2 | 0 | 0 | 2 | 2 | 2 | 2 | 0 | 1 | 2 | 1 | 2 | 16/24 |
| El Sallakh, 2012 | 2 | 1 | 0 | 2 | 2 | 2 | 2 | 0 | - | - | - | - | 11/16 |
| Saltzman, 2017 | 1 | 0 | 1 | 2 | 2 | 0 | 2 | 0 | - | - | - | - | 7/16 |
| Sammarco, 2002 | 0 | 0 | 1 | 2 | 1 | 1 | 2 | 0 | - | - | - | - | 6/16 |
| Savage-Elliott 2016 | 2 | 1 | 1 | 2 | 0 | 1 | 2 | 0 | - | - | - | - | 9/16 |
| Savva, 2007 | 2 | 2 | 1 | 2 | 0 | 2 | 0 | 0 | - | - | - | - | 8/16 |
| Sawa, 2008 | 2 | 2 | 1 | 2 | 2 | 1 | 2 | 0 | - | - | - | - | 11/16 |
| Schneider, 2009 | 1 | 0 | 0 | 2 | 1 | 1 | 2 | 0 | - | - | - | - | 7/16 |
| Shang, 2016 | 2 | 1 | 2 | 2 | 2 | 1 | 0 | 0 | 2 | 2 | 2 | 1 | 17/24 |
| Shimozono, 2017 | 2 | 0 | 2 | 2 | 2 | 2 | 2 | 0 | - | - | - | - | 12/16 |
| Shimozono, 2018 | 2 | 2 | 1 | 2 | 1 | 2 | 0 | 1 | 1 | 2 | 1 | 0 | 15/24 |
| Shimozono, 2018 | 2 | 1 | 1 | 2 | 1 | 1 | 2 | 2 | 1 | 2 | 0 | 1 | 16/24 |
| Shimozono, 2018 | 2 | 0 | 1 | 2 | 2 | 2 | 2 | 0 | - | - | - | - | 10/16 |
| Shimozono, 2019 | 2 | 1 | 1 | 2 | 1 | 1 | 0 | 1 | 1 | 2 | 1 | 0 | 13/24 |
| Tahta, 2017 | 2 | 1 | 1 | 2 | 1 | 2 | 1 | 0 | 2 | 2 | 1 | 1 | 16/24 |
| Takao, 2010 | 2 | 2 | 0 | 1 | 1 | 2 | 1 | 0 | - | - | - | - | 9/16 |
| Tao, 2014 | 2 | 0 | 0 | 2 | 2 | 1 | 2 | 0 | - | - | - | - | 10/16 |
| Taranow, 1999 | 2 | 0 | 1 | 2 | 2 | 2 | 2 | 0 | - | - | - | - | 11/16 |
| Usuelli, 2018 | 2 | 0 | 2 | 2 | 1 | 2 | 2 | 0 | - | - | - | - | 11/16 |
| Usuelli, 2016 | 2 | 2 | 0 | 2 | 2 | 2 | 2 | 0 | - | - | - | - | 12/16 |
| Usuelli, 2017 | 2 | 2 | 2 | 2 | 2 | 2 | 2 | 0 | - | - | - | - | 14/16 |
| Valderrabano, 2009 | 2 | 2 | 2 | 2 | 0 | 2 | 0 | 0 | - | - | - | - | 10/16 |
| Van Bergen, 2012 | 2 | 2 | 2 | 2 | 0 | 1 | 2 | 1 | - | - | - | - | 12/16 |
| Van Bergen, 2013 | 1 | 2 | 0 | 2 | 1 | 2 | 0 | 0 | - | - | - | - | 8/16 |
| Van Bergen, 2013 | 2 | 2 | 0 | 2 | 0 | 2 | 0 | 0 | - | - | - | - | 8/16 |
| Vannini, 2017 | 2 | 0 | 1 | 2 | 2 | 2 | 2 | 0 | - | - | - | - | 13/16 |
| Ventura, 2013 | 2 | 2 | 0 | 2 | 0 | 2 | 0 | 0 | - | - | - | - | 8/16 |
| Vira, 2017 | 2 | 0 | 1 | 2 | 2 | 0 | 2 | 0 | - | - | - | - | 9/16 |
| Vuurberg, 2018 | 2 | 2 | 2 | 2 | 0 | 2 | 0 | 1 | - | - | - | - | 11/16 |
| Walther, 2014 | 0 | 0 | 0 | 2 | 0 | 0 | 2 | 0 | - | - | - | - | 2/16 |
| Wiewiorski, 2013 | 2 | 0 | 0 | 2 | 2 | 1 | 2 | 0 | - | - | - | - | 9/16 |
| Yasui, 2014 | 2 | 0 | 1 | 2 | 2 | 2 | 2 | 0 | - | - | - | - | 11/16 |
| Yoshimura, 2015 | 2 | 0 | 1 | 2 | 2 | 2 | 2 | 0 | - | - | - | - | 11/16 |
| Yoshimura, 2013 | 1 | 0 | 0 | 2 | 1 | 1 | 2 | 0 | - | - | - | - | 7/16 |
| Zhu, 2016 | 2 | 2 | 0 | 2 | 0 | 2 | 0 | 0 | - | - | - | - | 8/16 |
| Acar, 2019 | 2 | 0 | 1 | 2 | 2 | 1 | 2 | 0 | - | - | - | - | 11/16 |
| Adanas, 2019 | 2 | 0 | 1 | 2 | 2 | 1 | 2 | 0 | - | - | - | - | 12/16 |
| Akpancar, 2019 | 2 | 0 | 1 | 2 | 2 | 0 | 2 | 0 | 0 | 2 | 2 | 0 | 13/24 |
| Kagan, 2019 | 2 | 2 | 1 | 2 | 2 | 1 | 2 | 0 | - | - | - | - | 12/16 |
| López-Alcorocho, 2019 | 2 | 2 | 2 | 2 | 2 | 1 | 2 | 0 | - | - | - | - | 13/16 |
| Haraguchi, 2019 | 2 | 0 | 1 | 2 | 2 | 1 | 2 | 0 | - | - | - | - | 10/16 |
| Jackson, 2019 | 2 | 0 | 0 | 2 | 1 | 1 | 2 | 0 | - | - | - | - | 8/16 |
| Lambers, 2019 | 2 | 0 | 0 | 2 | 2 | 2 | 2 | 0 | - | - | - | - | 10/16 |
| Haysbert, 2929 | 2 | 0 | 1 | 2 | 2 | 1 | 2 | 0 | - | - | - | - | 10/16 |
| Murphy, 2019 | 2 | 2 | 2 | 2 | 0 | 1 | 2 | 0 | - | - | - | - | 11/16 |
| Nakasa, 2019 | 2 | 0 | 0 | 2 | 2 | 1 | 2 | 0 | - | - | - | - | 9/16 |
| Nguyen, 2019 | 2 | 0 | 0 | 2 | 2 | 1 | 2 | 0 | - | - | - | - | 9/16 |
| Richter, 2019 | 2 | 2 | 2 | 2 | 2 | 2 | 2 | 0 | - | - | - | - | 14/16 |
| Richter, 2019 | 2 | 2 | 1 | 2 | 2 | 2 | 2 | 0 | 2 | 2 | 2 | 2 | 21/24 |
| Wang, 2019 | 2 | 0 | 1 | 2 | 2 | 2 | 2 | 0 | - | - | - | - | 11/16 |
| Weigelt, 2019 | 2 | 2 | 2 | 2 | 2 | 2 | 2 | 0 | - | - | - | - | 14/16 |
| Wei, 2019 | 2 | 0 | 0 | 2 | 2 | 2 | 2 | 0 | - | - | - | - | 10/16 |
| Yang, 2019 | 2 | 0 | 0 | 1 | 2 | 1 | 2 | 0 | - | - | - | - | 8/16 |
| Yontar, 2019 | 2 | 0 | 1 | 2 | 2 | 1 | 2 | 0 | - | - | - | - | 10/16 |
| Arıcan, 2019 | 2 | 0 | 1 | 2 | 2 | 1 | 2 | 0 | - | - | - | - | 10/16 |
| Basal, 2020 | 2 | 0 | 2 | 2 | 2 | 1 | 2 | 0 | - | - | - | - | 11/16 |
| Van Dijk, 2020 | 2 | 2 | 2 | 2 | 2 | 2 | 2 | 0 | - | - | - | - | 14/16 |
| Choi, 2020 | 2 | 2 | 2 | 2 | 2 | 2 | 2 | 0 | - | - | - | - | 14/16 |
| Heida, 2020 | 1 | 2 | 1 | 2 | 2 | 2 | 2 | 0 | - | - | - | - | 12/16 |
| Younger, 2020 | 2 | 0 | 2 | 1 | 2 | 1 | 2 | 0 | - | - | - | - | 10/16 |
| Mingqing, 2020 | 2 | 0 | 1 | 2 | 2 | 2 | 2 | 0 | - | - | - | - | 11/16 |
| Zhang, 2020 | 2 | 0 | 0 | 1 | 2 | 1 | 2 | 0 | - | - | - | - | 8/16 |
| Ahn, 2021 | 2 | 0 | 1 | 2 | 2 | 2 | 1 | 0 | - | - | - | - | 10/16 |
| Ayyaswamy, 2020 | 2 | 0 | 1 | 2 | 2 | 1 | 2 | 0 | - | - | - | - | 10/16 |
| Becher, 2019 | 2 | 0 | 1 | 2 | 2 | 2 | 2 | 0 | 2 | 2 | 2 | 2 | 19/24 |
| Casiri, 2020 | 2 | 0 | 1 | 2 | 2 | 2 | 2 | 0 | - | - | - | - | 11/16 |
| Camurcu, 2020 | 2 | 0 | 0 | 2 | 2 | 1 | 2 | 0 | - | - | - | - | 9/16 |
| Larsen, 2020 | 2 | 0 | 1 | 2 | 2 | 2 | 2 | 0 | - | - | - | - | 11/16 |
| Deng, 2020 | 2 | 0 | 1 | 2 | 2 | 0 | 2 | 0 | - | - | - | - | 9/16 |
| Drakos, 2021 | 2 | 0 | 2 | 1 | 2 | 1 | 2 | 0 | - | - | - | - | 10/16 |
| Hamilton, 2021 | 2 | 0 | 1 | 2 | 2 | 0 | 1 | 0 | - | - | - | - | 8/16 |
| Haradaa, 2020 | 2 | 0 | 0 | 2 | 2 | 0 | 2 | 0 | - | - | - | - | 8/16 |
| Yong, 2021 | 2 | 0 | 0 | 2 | 2 | 0 | 2 | 0 | - | - | - | - | 8/16 |
| Jungesblut, 2021 | 2 | 0 | 1 | 2 | 2 | 1 | 2 | 0 | - | - | - | - | 10/16 |
| Körner, 2021 | 2 | 0 | 1 | 2 | 2 | 2 | 2 | 0 | - | - | - | - | 11/16 |
| Lee , 2020 | 2 | 0 | 2 | 2 | 2 | 1 | 2 | 0 | 1 | 2 | 2 | 2 | 18/24 |
| Lenz, 2020 | 2 | 0 | 0 | 2 | 2 | 1 | 2 | 0 | - | - | - | - | 9/16 |
| Maioranoa, 2021 | 2 | 2 | 1 | 2 | 2 | 1 | 2 | 0 | - | - | - | - | 12/16 |
| Migliorini, 2021 | 2 | 2 | 2 | 2 | 2 | 1 | 2 | 0 | 2 | 2 | 2 | 1 | 20/24 |
| Minokawa, 2020 | 2 | 2 | 1 | 2 | 2 | 1 | 2 | 0 | - | - | - | - | 12/16 |
| Park, 2020 | 2 | 0 | 1 | 2 | 2 | 1 | 2 | 0 | - | - | - | - | 10/16 |
| Park, 2021 | 1 | 2 | 0 | 2 | 1 | 2 | 1 | 0 | - | - | - | - | 9/16 |
| Seo, 2018 | 2 | 2 | 1 | 2 | 2 | 2 | 1 | 0 | - | - | - | - | 12/16 |
| Pudda, 2020 | 2 | 0 | 1 | 2 | 2 | 1 | 2 | 0 | - | - | - | - | 10/16 |
| Choi, 2021 | 2 | 0 | 1 | 2 | 2 | 1 | 2 | 0 | - | - | - | - | 10/16 |
| Richter, 2020 | 2 | 0 | 0 | 1 | 2 | 0 | 2 | 0 | 1 | 2 | 2 | 1 | 13/24 |
| Sheu, 2021 | 2 | 0 | 1 | 2 | 2 | 0 | 2 | 0 | - | - | - | - | 9/16 |
| Shim, 2020 | 2 | 0 | 1 | 2 | 2 | 2 | 2 | 0 | 1 | 2 | 2 | 2 | 18/24 |
| Shimozono, 2020 | 2 | 0 | 1 | 2 | 2 | 1 | 2 | 0 | - | - | - | - | 10/16 |
| Toker, 2020 | 2 | 2 | 1 | 2 | 2 | 2 | 1 | 0 | - | - | - | - | 12/16 |
| Weigelt, 2020 | 2 | 0 | 1 | 2 | 2 | 2 | 2 | 0 | - | - | - | - | 11/16 |
| Abas,  2022 | 1 | 2 | 1 | 2 | 2 | 2 | 2 | 0 | - | - | - | - | 12/16 |
| Ackerman,  2021 | 2 | 2 | 1 | 2 | 1 | 2 | 1 | 0 | 2 | 2 | 2 | 2 | 19/24 |
| Allahabadi,  2021 | 2 | 2 | 1 | 2 | 1 | 2 | 2 | 0 | - | - | - | - | 12/16 |
| Azam,  2021 | 2 | 2 | 1 | 2 | 2 | 2 | 2 | 0 | 2 | 1 | 2 | 2 | 20/24 |
| Cassiri,  2021 | 1 | 2 | 1 | 2 | 2 | 2 | 2 | 0 | - | - | - | - | 12/16 |
| Dassari,  2021 | 2 | 2 | 1 | 2 | 1 | 2 | 1 | 0 | - | - | - | - | 11/16 |
| Danilkowicz, 2021 | 1 | 2 | 1 | 2 | 0 | 2 | 2 | 0 | 1 | 2 | 1 | 2 | 16/24 |
| L’Escalopier, 2021 | 1 | 2 | 1 | 2 | 2 | 2 | 2 | 0 | - | - | - | - | 12/16 |
| Dogar,  2021 | 1 | 2 | 2 | 2 | 0 | 2 | 2 | 0 | - | - | - | - | 11/16 |
| Geyer,  2021 | 1 | 2 | 1 | 1 | 0 | 2 | 2 | 0 | - | - | - | - | 9/16 |
| Gottschalk, 2021 | 1 | 2 | 2 | 2 | 1 | 1 | 2 | 0 | 2 | 2 | 1 | 2 | 18/24 |
| Gotze, 2021 | 2 | 2 | 2 | 2 | 1 | 2 | 1 | 0 | - | - | - | - | 12/16 |
| Hansen, 2021 | 2 | 2 | 1 | 2 | 1 | 2 | 2 | 0 | 2 | 2 | 2 | 2 | 20/24 |
| Kim, 2021 | 1 | 2 | 1 | 2 | 1 | 2 | 2 | 0 | 2 | 2 | 1 | 2 | 18/24 |
| Korner, 2021 | 2 | 2 | 1 | 2 | 1 | 2 | 2 | 0 | 2 | 2 | 2 | 2 | 20/24 |
| Li,  2021 | 2 | 2 | 1 | 1 | 2 | 2 | 2 | 0 | - | - | - | - | 12/16 |
| Mercer, 2022 | 2 | 2 | 1 | 2 | 2 | 2 | 2 | 0 | 2 | 1 | 2 | 2 | 20/24 |
| Ors,  2021 | 1 | 2 | 1 | 2 | 1 | 2 | 2 | 0 | - | - | - | - | 12/16 |
| Park,  2021 | 2 | 2 | 1 | 2 | 1 | 2 | 2 | 0 | - | - | - | - | 12/16 |
| Rikken, 2021 | 2 | 2 | 1 | 2 | 1 | 2 | 2 | 0 | 2 | 2 | 1 | 2 | 19/24 |
| Rizzo, 2021 | 2 | 2 | 2 | 2 | 2 | 2 | 2 | 0 | - | - | - | - | 14/16 |
| Schwartz, 2021 | 2 | 2 | 1 | 2 | 1 | 2 | 2 | 0 | 1 | 2 | 1 | 2 | 18/24 |
| Shim, 2021 | 2 | 2 | 1 | 2 | 2 | 2 | 2 | 0 | 2 | 2 | 1 | 2 | 20/24 |
| Shimozono . 2021 | 2 | 2 | 1 | 2 | 2 | 2 | 2 | 0 | 2 | 2 | 2 | 2 | 21/24 |
| Shi, 2022 | 2 | 2 | 1 | 2 | 1 | 2 | 2 | 0 | 2 | 2 | 2 | 2 | 20/24 |
| Vannini, 2021 | 2 | 2 | 1 | 2 | 1 | 2 | 1 | 0 | - | - | - | - | 11/16 |
| Viehofer, 2021 | 1 | 2 | 1 | 2 | 1 | 2 | 2 | 0 | - | - | - | - | 11/16 |
| Wang, 2022 | 1 | 2 | 1 | 2 | 2 | 2 | 2 | 0 | - | - | - | - | 12/16 |
| Wei, 2022 | 1 | 2 | 2 | 2 | 2 | 2 | 2 | 0 | 2 | 2 | 2 | 2 | 21/24 |
| Windhofer, 2022 | 1 | 2 | 1 | 2 | 1 | 2 | 1 | 0 | - | - | - | - | 10/16 |
| Zhang, 2021 | 1 | 2 | 1 | 2 | 1 | 1 | 2 | 0 | - | - | - | - | 10/16 |
| Zhang, 2022 | 1 | 2 | 1 | 2 | 2 | 2 | 2 | 0 | - | - | - | - | 12/16 |
| Guo, 2022 | 2 | 2 | 1 | 2 | 2 | 2 | 2 | 0 | - | - | - | - | 13/16 |
| Gianakos, 2023 | 2 | 2 | 1 | 2 | 2 | 2 | 2 | 1 | - | - | - | - | 14/16 |
| Gianakos,  2023 | 2 | 2 | 1 | 2 | 2 | 2 | 1 | 0 | - | - | - | - | 12/16 |
| Rikken, 2022 | 2 | 2 | 2 | 2 | 2 | 2 | 2 | 2 | - | - | - | - | 16/16 |
| Saw, 2022 | 1 | 1 | 1 | 2 | 2 | 2 | 2 | 0 | - | - | - | - | 10/16 |
| Bai, 2022 | 2 | 1 | 1 | 2 | 2 | 2 | 2 | 0 | - | - | - | - | 12/16 |
| Tan, 2022 | 2 | 2 | 1 | 2 | 2 | 2 | 2 | 0 | 2 | 2 | 1 | 2 | 20/24 |
| Schafer, 2022 | 2 | 2 | 1 | 2 | 2 | 2 | 2 | 0 | - | - | - | - | 13/16 |
| Aldahshan, 2022 | 2 | 2 | 2 | 2 | 2 | 1 | 2 | 0 | - | - | - | - | 13/16 |
| Richter, 2022 | 2 | 2 | 2 | 2 | 2 | 2 | 2 | 2 | - | - | - | - | 16/16 |
| Winkler, 2022 | 2 | 2 | 1 | 2 | 2 | 2 | 2 | 0 | - | - | - | - | 13/16 |
| Kim, 2022 | 2 | 2 | 1 | 2 | 2 | 1 | 2 | 0 | - | - | - | - | 12/16 |
| Fu, 2022 | 2 | 1 | 1 | 2 | 2 | 2 | 2 | 0 | - | - | - | - | 12/16 |
| Migliorini, 2022 | 1 | 1 | 1 | 2 | 2 | 1 | 2 | 0 | - | - | - | - | 10/16 |
| Choi, 2022 | 2 | 1 | 1 | 2 | 2 | 1 | 2 | 0 | - | - | - | - | 11/16 |
| Drakos, 2022 | 1 | 2 | 1 | 2 | 2 | 2 | 2 | 0 | - | - | - | - | 12/16 |
| Windhofer, 2022 | 2 | 2 | 1 | 2 | 2 | 1 | 2 | 0 | - | - | - | - | 12/16 |
| Zhao, 2022 | 2 | 2 | 1 | 2 | 2 | 2 | 2 | 0 | - | - | - | - | 13/16 |
| Fletcher, 2022 | 2 | 2 | 2 | 2 | 2 | 2 | 2 | 0 | - | - | - | - | 14/16 |
| Saxena, 2022 | 2 | 2 | 2 | 2 | 2 | 2 | 2 | 0 | - | - | - | - | 14/16 |
| Yontar, 2022 | 2 | 2 | 1 | 2 | 2 | 2 | 2 | 0 | - | - | - | - | 13/16 |
| Nakasa, 2022 | 2 | 2 | 1 | 2 | 2 | 1 | 2 | 0 | 1 | 2 | 1 | 2 | 18/24 |
| de l'Escalopier, 2021 | 2 | 2 | 1 | 2 | 2 | 2 | 2 | 0 | - | - | - | - | 13/16 |
| Wan, 2022 | 2 | 2 | 1 | 2 | 2 | 1 | 2 | 0 | - | - | - | - | 12/16 |
| Ahrend, 2022 | 2 | 1 | 1 | 2 | 2 | 2 | 2 | 0 | - | - | - | - | 12/16 |
| Geyer, 2022 | 2 | 1 | 1 | 2 | 2 | 1 | 2 | 0 | - | - | - | - | 11/16 |
| Allahabadi, 2022 | 2 | 2 | 1 | 2 | 2 | 2 | 2 | 0 | - | - | - | - | 13/16 |
| Zhang, 2022 | 2 | 2 | 1 | 2 | 2 | 1 | 2 | 0 | 2 | 2 | 2 | 2 | 19/24 |
